# Triptolide promotes autophagy to inhibit mesangial cell proliferation in IgA nephropathy via the CARD9/p38 MAPK pathway

**DOI:** 10.1111/cpr.13278

**Published:** 2022-06-22

**Authors:** Lu Zhao, Zhixin Lan, Liang Peng, Lili Wan, Di Liu, Xia Tan, Chengyuan Tang, Guochun Chen, Hong Liu

**Affiliations:** ^1^ Department of Nephrology The Second Xiangya Hospital of Central South University, Hunan Key Laboratory of Kidney Disease and Blood Purification Changsha Hunan China

## Abstract

**Background:**

Mesangial cell proliferation is the most basic pathological feature of immunoglobulin A nephropathy (IgAN); however, the specific underlying mechanism and an appropriate therapeutic strategy are yet to be unearthed. This study aimed to investigate the therapeutic effect of triptolide (TP) on IgAN and the mechanism by which TP regulates autophagy and proliferation of mesangial cells through the CARD9/p38 MAPK pathway.

**Methods:**

We established a TP‐treated IgAN mouse model and produced IgA1‐induced human mesangial cells (HMC) and divided them into control, TP, IgAN, and IgAN+TP groups. The levels of mesangial cell proliferation (PCNA, cyclin D1, cell viability, and cell cycle) and autophagy (P62, LC3 II, and autophagy flux rate) were measured, with the autophagy inhibitor 3‐Methyladenine used to explore the relationship between autophagy and proliferation. We observed CARD9 expression in renal biopsies from patients and analyzed its clinical significance. CARD9 siRNA and overexpression plasmids were constructed to investigate the changes in mesangial cell proliferation and autophagy as well as the expression of CARD9 and p‐p38 MAPK/p38 MAPK following TP treatment.

**Results:**

Administering TP was safe and effectively alleviated mesangial cell proliferation in IgAN mice. Moreover, TP inhibited IgA1‐induced HMC proliferation by promoting autophagy. The high expression of CARD9 in IgAN patients was positively correlated with the severity of HMC proliferation. CARD9/p38 MAPK was involved in the regulation of HMC autophagy and proliferation, and TP promoted autophagy to inhibit HMC proliferation by downregulating the CARD9/p38 MAPK pathway in IgAN.

**Conclusion:**

TP promotes autophagy to inhibit mesangial cell proliferation in IgAN via the CARD9/p38 MAPK pathway.

## Graphical abstract

The proliferation of glomerular mesangial cells is the most basic pathological feature for IgA nephropathy (IgAN). Galactose‐deficient IgA1 (Gd‐IgA1) immune complex deposition activates mesangial cells to mediate specific intracellular signal transduction, promotes mesangial cell proliferation, and initiates kidney damage. Autophagy is involved in mesangial cell proliferation. CARD9 is a risk gene for IgAN. The triptolide (TP), purified from *Tripterygium wilfordii* hook F., promotes autophagy to inhibit mesangial cell proliferation in IgAN via the CARD9/p38 MAPK pathway.

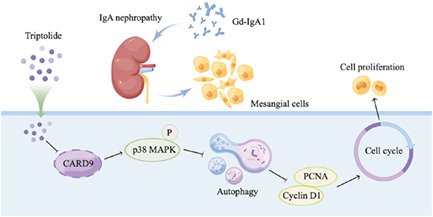

## 1 INTRODUCTION

IgA nephropathy (IgAN) is a primary glomerulonephritis characterized by the glomerular mesangial deposition of IgA or IgA‐based immunoglobulin.^1^ IgAN is the most common primary glomerular disease worldwide and accounts for 40–50% of such diseases in Asia.^2^, ^3^ Currently, a specific therapy has not been developed for IgAN, and patients are managed primarily with optimized supportive treatment.^4^ Moreover, approximately 30–40% of IgAN patients will eventually progress to end‐stage kidney disease (ESKD).^5^

The proliferation of glomerular mesangial cells and extracellular matrix is the most basic pathological feature for IgAN.^6^, ^7^ Galactose‐deficient IgA1 (Gd‐IgA1) immune complex deposition activates mesangial cells to mediate specific intracellular signal transduction, promotes mesangial cell proliferation, and initiates kidney damage. Moreover, mesangial cells release a series of pro‐inflammatory and fibroblast cytokines to mediate oxidative stress and complement activation, thereby leading to podocyte and proximal tubular epithelial cell damage, glomerular sclerosis, and interstitial fibrosis, which together promote IgAN progression.^8^, ^9^, ^10^ These studies suggest that mesangial cells play a key role in the early pathological changes in IgAN; however, the regulatory mechanism remains unclear.

Our previous studies showed that autophagy is involved in IgAN pathogenesis.^11^, ^12^ Rapamycin and dihydroartemisinin treatment inhibited the mTOR/S6k1 pathway to enhance autophagy and reduce mesangial cell proliferation. Autophagy is a process of engulfing the cell's own cytoplasm and organelles that are in the form of vesicles by fusing these vesicles with lysosomes, forming autolysosomes that degrade their contents.^13^ Autophagy is crucial for maintaining kidney cell function and homeostasis,^14^, ^15^ and autophagy activation could be a potential therapy for kidney disease; however, few related studies have focused on IgAN.^16^

*Tripterygium wilfordii* hook F. (TwHF) is a traditional Chinese herbal medicine with immunosuppressive and anti‐inflammatory effects, and it is widely used in the treatment of cancer, inflammation, and autoimmune rheumatic disease.^17^, ^18^, ^19^ Since 1977, reports have shown that it can reduce hematuria, proteinuria, and edema in various kidney diseases.^20^, ^21^, ^22^ However, the composition of TwHF is complicated.^23^, ^24^ We previously found that triptolide (TP) was the main active ingredient of TwHF according to a “compound‐targets‐disease” analysis based on network pharmacology.^25^ The potency of TP is 100–200‐times higher than that of tripterygium glycosides, and it shows improved bioavailability and reduced side effects.^26^, ^27^ In various diseases, TP regulates cell proliferation, apoptosis, differentiation, invasion, and immune responses by inducing autophagy.^28^, ^29^, ^30^, ^31^, ^32^, ^33^ However, the exact mechanism of action of TP in IgAN remains unclear.

A genome‐wide association study (GWAS) showed that caspase recruitment domain‐containing protein 9 (CARD9) is a risk gene for IgAN.^34^, ^35^ The single nucleotide polymorphism rs4077515 of the CARD9 coding sequence is significantly related to a decline in renal function and promotes glomerular inflammation induced by IgA deposition.^36^ However, the specific role of CARD9 in IgAN pathogenesis has not been investigated. CARD9 is an intracellular adaptor protein of the CARD family. It is a downstream effector molecule of pattern recognition receptors (PRRs), including pathogen associated molecular patterns (PAMPs) and damage associated molecular patterns (DAMPs), which are involved in the regulation of immune homeostasis, inflammatory responses, autophagy, and proliferation in infections, inflammation, tumors, and autoimmune diseases.^37^, ^38^, ^39^ CARD9 is a potential target for the treatment of cardiovascular diseases by regulating autophagy. Studies have reported that high expression of CARD9 in transverse aortic coarctation inhibits autophagy of cardiomyocytes, thus leading to pathological cardiac remodeling.^40^ CARD9‐knockout obese mice showed enhanced autophagy by reducing p38 MAPK phosphorylation to improve obesity‐related myocardial dysfunction.^41^ p38 MAPK is a transcription factor that is regulated by CARD9 and has been shown to inhibit autophagy in various diseases.^42^, ^43^, ^44^, ^45^, ^46^, ^47^, ^48^, ^49^, ^50^, ^51^

In this study, we investigated the roles of TP and CARD9 in IgAN and the mechanisms underlying the associated autophagy and mesangial cell proliferation. We speculated that TP promotes autophagy via the CARD9/p38 MAPK pathway to inhibit mesangial cell proliferation, thereby improving IgAN.

## 2 MATERIALS AND METHODS

### 2.1 Animals

Twenty‐six five‐week‐old female BALB/c mice weighing 20–25 g were obtained from Hunan Slack Jingda Experimental Animal Co. Ltd. (Changsha, China). All animal procedures were approved by the Experimental Animal Ethics Committee of the Second Xiangya Hospital of the Central South University. After 7 days of adaptive pre‐feeding, the mice were randomly divided into four groups: control, TP, IgAN, and IgAN+TP. The IgAN mouse model was established by oral mucosal immunization. Gavage with acidified bovine serum albumin (BSA) was performed once every other day (800 mg/kg), and the mixed solution (CCL4: castor oil = 1:5) was subcutaneously injected weekly (0.1 ml) and intraperitoneally injected every 2 weeks (0.06–0.08 ml). Lipopolysaccharide (50 μg) was injected into the tail vein at weeks 6 and 8 week. The IgAN mouse model was successfully established at the end of the 11th week, as confirmed by glomerular IgA immunofluorescence. The TP and IgAN+TP groups received intragastric TP treatment (300 μg/kg/day). The control and IgAN groups were administered the same dose of normal saline. At the end of the 17th week, mice were sacrificed by cervical dislocation. Blood, urine, and kidney tissue samples were collected for the subsequent experiments.

According to the manufacturer's standards, the serum alanine aminotransferase (ALT), aspartate aminotransferase (AST), albumin (ALB), serum creatinine (SCr), blood urea nitrogen (BUN), and uric acid (UA) were detected with enzyme‐linked immunosorbent (ELISA) assay kits (Leidu, China). The urine protein and creatinine levels of the mice were detected using ELISA kits (Jiancheng, China), and the ratios of urine protein/creatinine were calculated.

### 2.2 Cell culture and treatment

The human glomerular mesangial cell line (HMC) (CBR130735, Cellbio, China) was cultured in DMEM/F‐12 medium (Gibco, USA) with 10% fetal bovine serum (FBS) and 1% antibiotics at 37 °C and 5% CO2. As previously described, monomeric human IgA1 (Abcam, USA) was heated at 65 °C for 150 min to polymerize into polymer IgA1.^52^ The cells were incubated with IgA1 (25 μg/ml) for 24 h to construct an in vitro cell model of IgAN. TP or the autophagy inhibitor 3‐Methyladenine (3‐MA) (Selleck, China) was soluble in dimethyl sulfoxide (DMSO). Cells were incubated with TP (20 ng/ml) (Figure S1) and/or 3‐MA (5 mM) for 24 h with or without IgA1.

### 2.3 Transfection of siRNA and plasmid

The sequence of the CARD9‐siRNA (siCARD9) (Riobio, China) was 5′‐CCTCACGCATCACACCTTA‐3′. The CARD9‐overexpression plasmid (Exp‐CARD9) (Genechem, China) was designed according to the GenBank reference sequence (NM_052813). Lipofectamine 2000 (Invitrogen, USA) was used to transfect CARD9‐siRNA or overexpression plasmid into HMCs according to the manufacturer's instructions.

### 2.4 Patients

All patients were diagnosed based on renal biopsy at the Second Xiangya Hospital of Central South University. All biopsies were obtained from the kidney pathology laboratory of the hospital, and clinical information was obtained from the electronic medical records. All procedures were performed in accordance with the Declaration of Helsinki and were approved by the Ethics Committee of the Second Xiangya Hospital of Central South University. Thirty patients with IgAN were enrolled, and mesangial cell proliferation was assessed based on pathological diagnosis reports (normal: mesangial cells <4/mesangial area; mild: 4–5/area; moderate: 6–7/area; severe: ≥8/area).

### 2.5 Histology and immunohistochemical (IHC) staining

Kidney tissues were fixed in 4% paraformaldehyde and embedded in paraffin. Hematoxylin–eosin (H&E) and periodic acid‐Schiff (PAS) staining was performed to observe the proliferation of glomerular mesangial cells. According to the instructions of the immunohistochemistry kit (ZSGB‐Bio, China), the expressions of cyclin D1 (1:100, Abcam, USA), PCNA, P62 (1:100, Proteintech, China), and LC3 II (1:100, Servicebio, China) were detected. Quantitative analysis was performed using ImageJ software.

### 2.6 Immunofluorescence

Immunofluorescence analysis of mouse IgA was performed as previously described.^53^ Double IF of the renal biopsies was performed as follows. Three micrometer‐thick paraffin‐embedded sections were deparaffinized with a series of xylene and ethanol and then rehydrated. Proteinase K (50 ng/ml, Beyotime, China) was used for antigen retrieval for 30 min at room temperature, followed by blocking with QuickBlockTM Blocking Buffer (Beyotime, China) at room temperature for 15 min. Subsequently, incubation with CARD9 (1:50, Santa Cruz, USA) and anti‐human Gd‐IgA1 (KM55) rat IgG (100 mg/ml, IBL, Japan) was performed overnight at 4 °C. Next, goat anti‐rat IgG antibody (1:50; Life Technologies, USA) conjugated with Alexa Fluor 555 and goat anti‐mouse IgG conjugated with Alexa Fluor 488 (1:1000; Abcam, USA) were incubated at 37 °C for 2 h. A drop of anti‐fading DAPI (Servicebio) was used to seal the samples. We observed Gd‐IgA1 and CARD9 under a fluorescence microscope (Leica, Germany) and performed colocalization analyses using ImageJ. For HMCs, PCNA (1:100, Abcam, USA), P62 (1:50, Proteintech, China) and cyclin D1 (1:50, Abcam, USA) were used. The cells were washed with phosphate‐buffered saline, fixed with 4% paraformaldehyde, and permeabilized with 0.1% Triton X‐100. Alexa Fluor 594 conjugated goat anti‐rabbit IgG (1:1000; Abcam, USA) was used as the secondary antibody. The remaining procedures were performed as previously described.

### 2.7 Quantitative reverse transcription PCR analysis

Total RNA was extracted from cells using TRIzol reagent (Invitrogen, USA). The HiScript III RT Supermix (+gDNA wiper) (Vazyme, China) was used for mRNA reverse transcription. Real‐time PCR was performed on a LightCycler 96 System (Roche, Germany) using the SYBR Green Master Mix (Servicebio, China). The primer sequences were CARD9: F‐CAGCCCCTACATCCAGGTA and R‐CAGGGAGAAGATGGTGTTGG. CARD9 and internal reference primers were purchased from Sangon Company (China).

### 2.8 Western blotting

Radioimmunoprecipitation assay lysis buffer containing phenylmethylsulfonyl fluoride and phosphatase inhibitors was used to extract cell and tissue proteins. Equal amounts of protein samples were loaded on SDS‐PAGE and transferred to polyvinylidene fluoride membranes. The membranes were blocked in 5% BSA at room temperature for 1 h, followed by incubation overnight at 4°C with the following primary antibodies: cyclin D1 (1:10000, Abcam, USA), PCNA, P62 (1:1000, Proteintech, China), LC3 II (1:500, Servicebio, China), β‐actin (1:5000, ProteinFind, China), GAPDH (1:1000, Proteintech, China), CARD9 (1:1000, Abcam, USA), p‐p38 MAPK (1:1000, Cell Signaling Technology, USA), p38 MAPK (1:1000, Cell Signaling Technology, USA). Membranes were then incubated with secondary antibodies conjugated to horseradish peroxidase. Chemiluminescence signals were identified using ECL kits (Advansta, USA) and quantitatively analyzed using ImageJ.

### 2.9 Cell proliferation and cell cycle assay

A Cell Counting Kit‐8 (CCK‐8) assay (Beyotime, China) was performed to detect cell viability, and the number of live cells was estimated by measuring the optical density (OD) at 450 nm. Cell cycle distribution was analyzed using flow cytometry (Becton Dickinson, USA). The proliferation index (PI) was calculated as the sum of the percentages of cells in the S and G2/M phases. Cells were fixed with 75% ice‐cold ethanol at 4 °C overnight and added propidium iodide and RNase A (Beyotime, China) mixed working solution, followed by staining for 30 min at 4 °C in the dark. FlowJo V10 was used to process and analyze the data from the flow cytometer.

### 2.10 Autophagy dynamics analysis

As mentioned in previous reports, the dynamic process of autophagy was analyzed in cells expressing mRFP‐GFP‐LC3.^54^, ^55^ The acid‐sensitive GFP was quenched in a low‐pH lysosomal environment, while the acid‐insensitive RFP was more stable. Therefore, the colocalization of RFP and GFP together indicated autophagosomes while the localization of RFP alone indicated autophagolysosomes. HMCs were transiently transfected with mRFP‐GFP‐LC3 (ptfLC3; Addgene plasmid 21074) and fixed with 4% paraformaldehyde. For the quantitative analysis, approximately 10 random cells (×630) were analyzed for each condition using a confocal laser scanning microscope (BIO‐ROD1024, UK). ImageJ software was used to calculate the number of GFP‐LC3 and RFP‐LC3 puncta per cell. The number of autophagosomes is represented by merged yellow puncta, and the number of autophagolysosomes is represented by subtracting GFP puncta from RFP puncta. The autophagy flux rate was calculated by dividing the number of autolysosomes by the total number of RFP puncta.

### 2.11 Statistical analysis

Data are presented as the mean ± SD. Two‐tailed Student's *t*‐test was used to determine the statistical difference between the two groups, and one‐way analysis of variance was used to analyze the differences between multiple groups. Bivariate correlation analysis was adopted for the two variables. All experiments were independently repeated at least three times, and statistical significance was set at *p* < 0.05. All statistical analyses were performed using GraphPad Prism 8.0 and SPSS 26.0 software.

## 3 RESULTS

### 3.1 Effects of TP on mesangial cell proliferation and autophagy in IgAN mice

**FIGURE 1:**
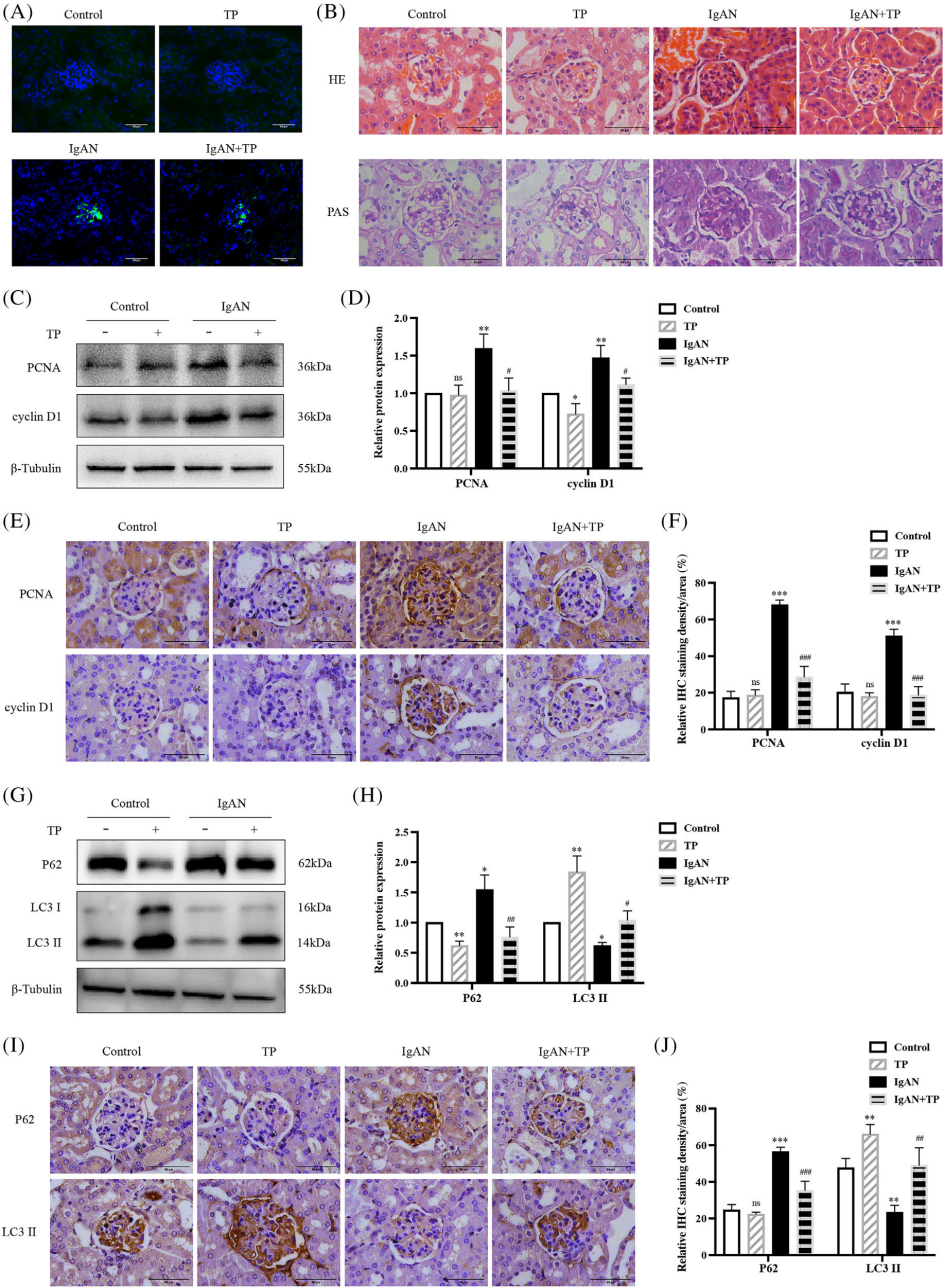
TP inhibits mesangial cell proliferation and promotes autophagy in IgAN mice. (A) Immunofluorescence of IgA in glomeruli. (B) HE and PAS staining. Representative western blots (C) and quantitative analysis (D) of PCNA and cyclin D1 in renal cortex. IHC staining (E) and quantitative analysis (F) of PCNA and cyclin D1 in glomeruli. Representative western blots (G) and quantitative analysis (H) of P62 and LC3 II in renal cortex. IHC staining (I) and quantitative analysis (J) of P62 and LC3 II in glomeruli. All data presented as mean ± SD, *n* = 3. ns: *p* > 0.05 vs. Control, *p < 0.05, **p < 0.01, ***p < 0.001 vs. Control, ^#^ *p* < 0.05, ^##^ *p* < 0.01, ^###^ *p* < 0.001 vs. IgAN. Bars = 50 μm; All images ×400.

First, we established a TP‐treated IgAN mouse model. IF showed that IgA was significantly deposited in the mesangium of IgAN mice compared to that in control mice (Figure 1A). H&E and PAS staining showed increased proliferation of IgAN mesangial cells (Figure 1B). After TP treatment, mesangial IgA deposition and cell proliferation were significantly reduced in the IgAN+TP mice. WB and IHC showed high expression of PCNA and cyclin D1 in IgAN mice, especially in the mesangium, indicating increased mesangial cell proliferation (Figure 1C–F). Meanwhile, high P62 and low LC3 II levels indicated that autophagy in IgAN mesangial cells was suppressed (Figure 1G–J). In IgAN+TP mice, PCNA, cyclin D1, and P62 decreased while LC3 II increased, indicating that TP effectively inhibited mesangial cell proliferation and promoted autophagy in IgAN mice (Figure 1C–J). Table 1 shows the hepatorenal function and urine PCR results of mice. The ALB, BUN, and SCr levels declined slightly after TP treatment but remained within the normal range, indicating that TP was safe.

**TABLE 1:** Characteristics of TP‐treated IgAN mice

	Control (*n* = 6)	TP (*n* = 6)	IgAN (*n* = 6)	IgAN+TP (*n* = 6)
ALT (U/L)	42.7 ± 1.4	32.2 ± 5.9	73.7 ± 31.1*	61.5 ± 11.6
AST (U/L)	102.6 ± 2.0	303.5 ± 165.7	139.6 ± 12.0	271.5 ± 141.6
ALB (g/L)	32.3 ± 2.0	22.1 ± 2.3**	35.1 ± 2.8	24.4 ± 4.6*^##^
BUN (mg/dl)	22.7 ± 3.1	15.4 ± 2.3*	24.7 ± 5.6	16.3 ± 3.3*^#^
SCr (μmol/L)	21.4 ± 2.1	16.0 ± 4.6	27.1 ± 8.2	13.9 ± 3.9^#^
UA (μmol/L)	144.2 ± 78.0	186.5 ± 28.2	192.8 ± 98.5	253.1 ± 65.8
PCR (mg/g)	945.3 ± 64.7	1372.6 ± 740.6	1788.3 ± 1036.5	1046.7 ± 433.8

*Note*: *p < 0.05, **p < 0.01 vs. Control; ^#^ *p* < 0.05, ^##^ *p* < 0.01 vs. IgAN. Normal reference ranges: ALT (10.06–96.47 U/L); AST (36.31–235.48 U/L); ALB (21.22–39.15 g/L); BUN (10.81–34.74 mg/dl); SCr (10.91–85.09 μmol/L); and UA (44.42–224.77 μmol/L). All data presented as mean ± SD, *n* = 6.

Abbreviations: ALB, albumin; ALT, alanine aminotransferase; AST, aspartate aminotransferase; BUN, blood urea nitrogen; PCR, ratios of urine protein/creatinine; SCr, serum creatinine; UA, uric acid.

### 3.2 Effects of TP on proliferation and autophagy of IgA1‐induced HMCs

**FIGURE 2:**
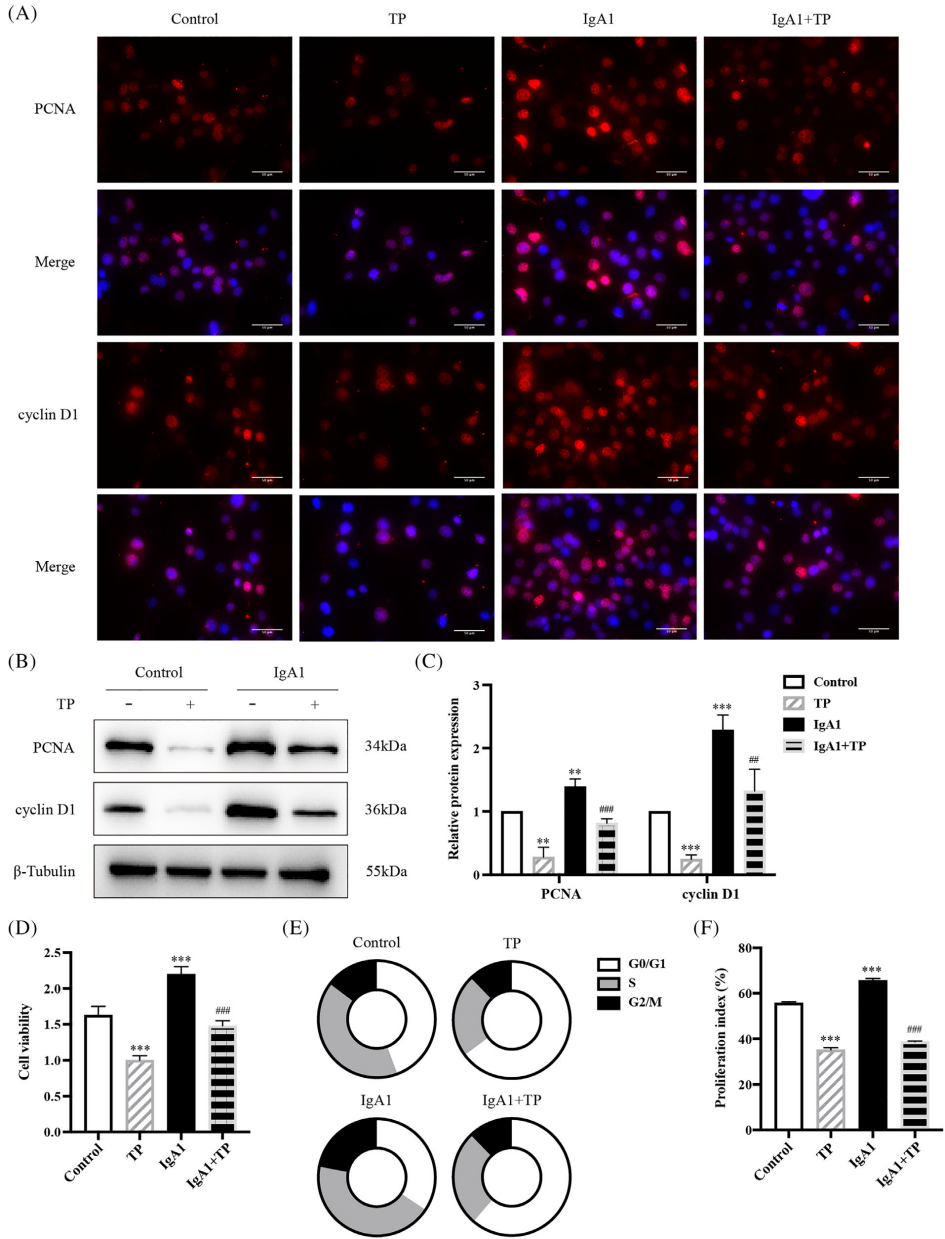
TP inhibits IgA1‐induced HMC proliferation. (A) Immunofluorescence of PCNA and cyclin D1 in HMCs. Bars = 50 μm, All images ×400. Representative western blots (B) and quantitative analysis (C) of PCNA and cyclin D1 in HMCs. (D) Cell viability was assessed by CCK8 assay. (E,F) Flow cytometry analysis of cell cycle in HMCs. The proliferation index is calculated as the sum of the percentages of cells in the S phase and G2/M phase. All data presented as mean ± SD, *n* = 3. *p < 0.05, **p < 0.01, ***p < 0.001 vs. Control, ^#^ *p* < 0.05, ^##^ *p* < 0.01, ^###^ *p* < 0.001 vs. IgA1.

We also investigated the effects of TP on HMC proliferation and autophagy. IF and WB showed that high expression of PCNA and cyclin D1 in IgA1‐induced HMCs was reduced by TP (Figure 2A–C). The CCK‐8 assay indicated that IgA1 promoted cell viability while TP inhibited this effect (Figure 2D). Cell cycle analysis by flow cytometry showed that IgA1 stimulation increased the percentages of cells in the S and G2/M phases, while TP arrested cells at the G0/G1 phase (Figure 2E). TP reduced the proliferation index of IgA1‐induced HMCs (Figure 2F). These results indicated that TP inhibited IgA1‐induced HMC proliferation.

**FIGURE 3:**
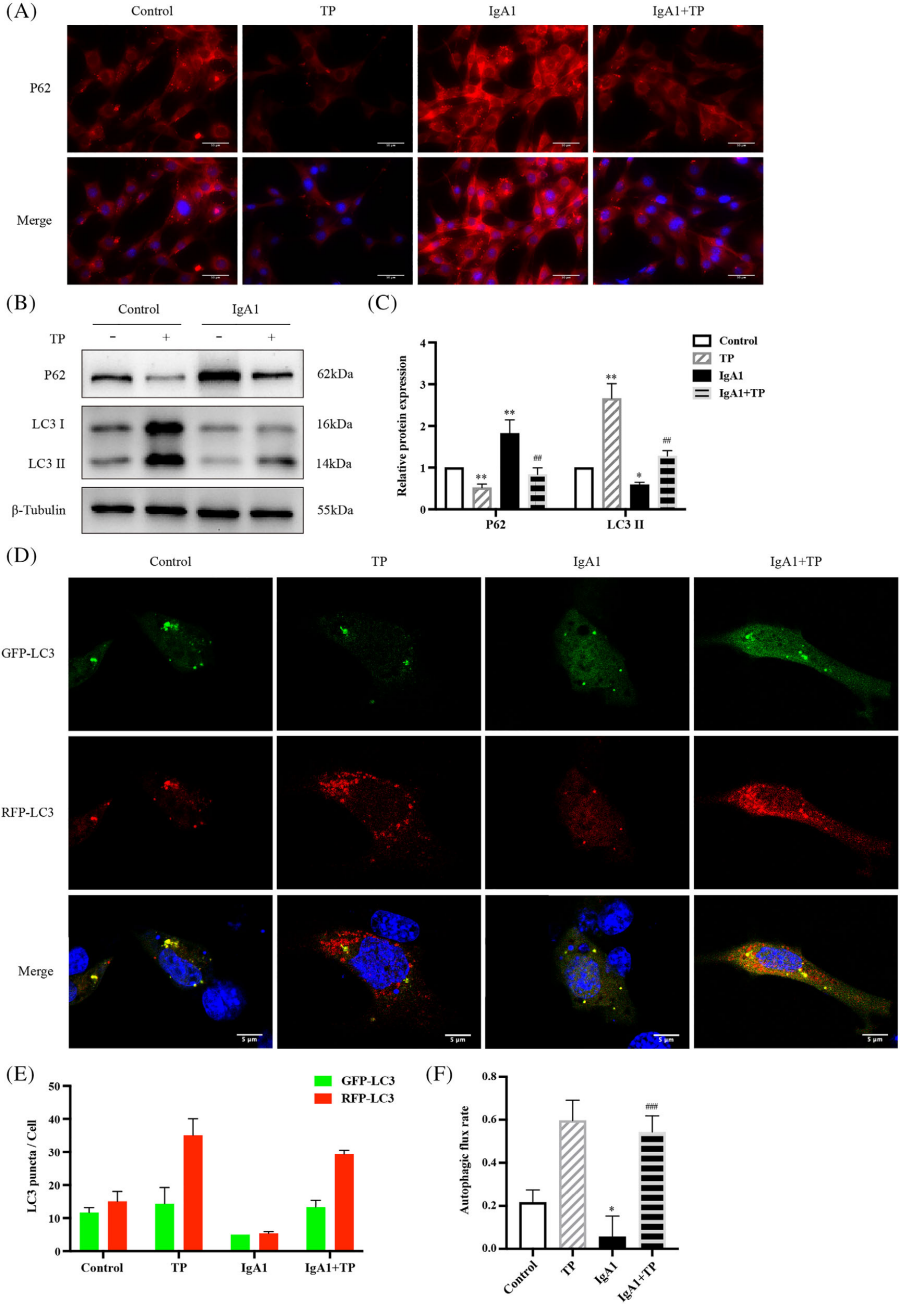
TP promotes autophagy in IgA1‐induced HMC. (A) Immunofluorescence of P62 in HMCs. Bars = 50 μm, All images ×400. Representative western blots (B) and quantitative analysis (C) of P62 and LC3 II in HMCs. (D) Representative images of GFP‐LC3 and RFP‐LC3 puncta in transfected HMCs. Bars = 5 μm, All images ×630. (E) Quantitative analysis of GFP‐LC3 and RFP‐LC3 puncta. (F) Analysis of autophagic flux rate. All data presented as mean ± SD, *n* = 3. *p < 0.05, **p < 0.01 vs. Control, ^##^ *p* < 0.01, ^###^ *p* < 0.001 vs. IgA1.

As shown in Figure 3, P62 was increased and LC3 II expression was decreased in IgA1‐induced HMCs. TP downregulated P62 and upregulated LC3 II (Figure 3A–C). Furthermore, we observed HMCs transfected with mRFP‐GFP‐LC3 plasmid using a confocal laser scanning microscope (Figure 3D). The yellow puncta of GFP and RFP colocalization represent autophagosomes, and the red puncta of RFP alone represent autolysosomes. In IgA1‐induced HMCs, the average number of autophagosomes and autolysosomes was 5 and 5.3, respectively, whereas after TP treatment, the average number of autophagosomes and autolysosomes increased to 13.3 and 29.3, respectively (Figure 3E). We quantitatively evaluated the autophagic flux rate by calculating the ratio of autolysosomes to total RFP puncta (Figure 3F). Compared with the basal rate of 20% in the control HMCs, the autophagic flux rate was reduced to 6% in IgA1‐induced HMCs. Moreover, TP increased by 54%. These results indicated that TP promoted autophagy in IgA1‐induced HMCs.

### 3.3 TP inhibited IgA1‐induced HMC proliferation by promoting autophagy

**FIGURE 4:**
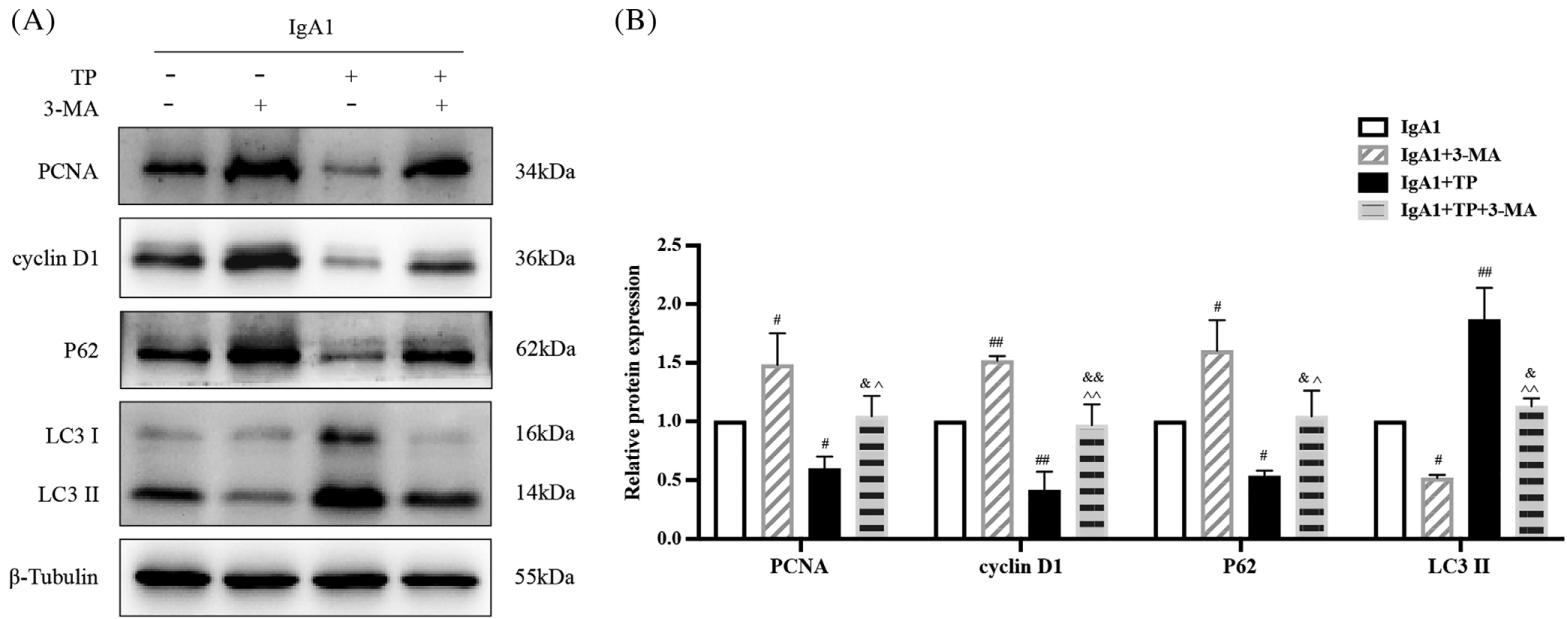
TP inhibits HMC proliferation by promoting autophagy. Representative western blots (A) and quantitative analysis (B) of PCNA, cyclin D1, P62, and LC3 II in IgA1‐induced HMCs under TP or 3‐MA conditions. All data presented as mean ± SD, *n* = 3. ^#^ *p* < 0.05, ^##^ *p* < 0.01 vs. IgA1; ^&^ *p* < 0.05, ^&&^ *p* < 0.01 vs. IgA1 + 3‐MA; ^^^ *p* < 0.05, ^^^^ *p* < 0.01 vs. IgA1 + TP.

We used the autophagy inhibitor 3‐MA to explore the relationship between autophagy and proliferation under IgA1 and TP conditions (Figure 4A,B). 3‐MA further increased PCNA, cyclin D1, and P62 and decreased LC3 II in IgA1‐induced HMCs, indicating that 3‐MA effectively suppressed autophagy and promoted HMC proliferation. Importantly, the antiproliferative effects of TP were inhibited by 3‐MA. The IgA1 + TP + 3‐MA group showed upregulated levels of PCNA, cyclin D1, and P62 but downregulated levels of LC3 II compared to that in the IgA1 + TP group. In summary, TP inhibits HMC proliferation by promoting autophagy in IgAN.

### 3.4 CARD9 positively correlated with IgAN mesangial proliferation

We used the dataset GSE116626 (11 control and 37 IgAN samples) from the GEO database to analyze differentially expressed genes in human kidney biopsy tissues. CARD9 was among the top 30 differentially expressed genes (Figure S2A). The level of CARD9 mRNA in patients with IgAN was significantly increased (*p* < 0.05, log2 [FC] = 0.7) (Figure S2B). Notably, a GWAS indicated that CARD9 is an IgAN risk gene; therefore, we focused on its role.

**FIGURE 5:**
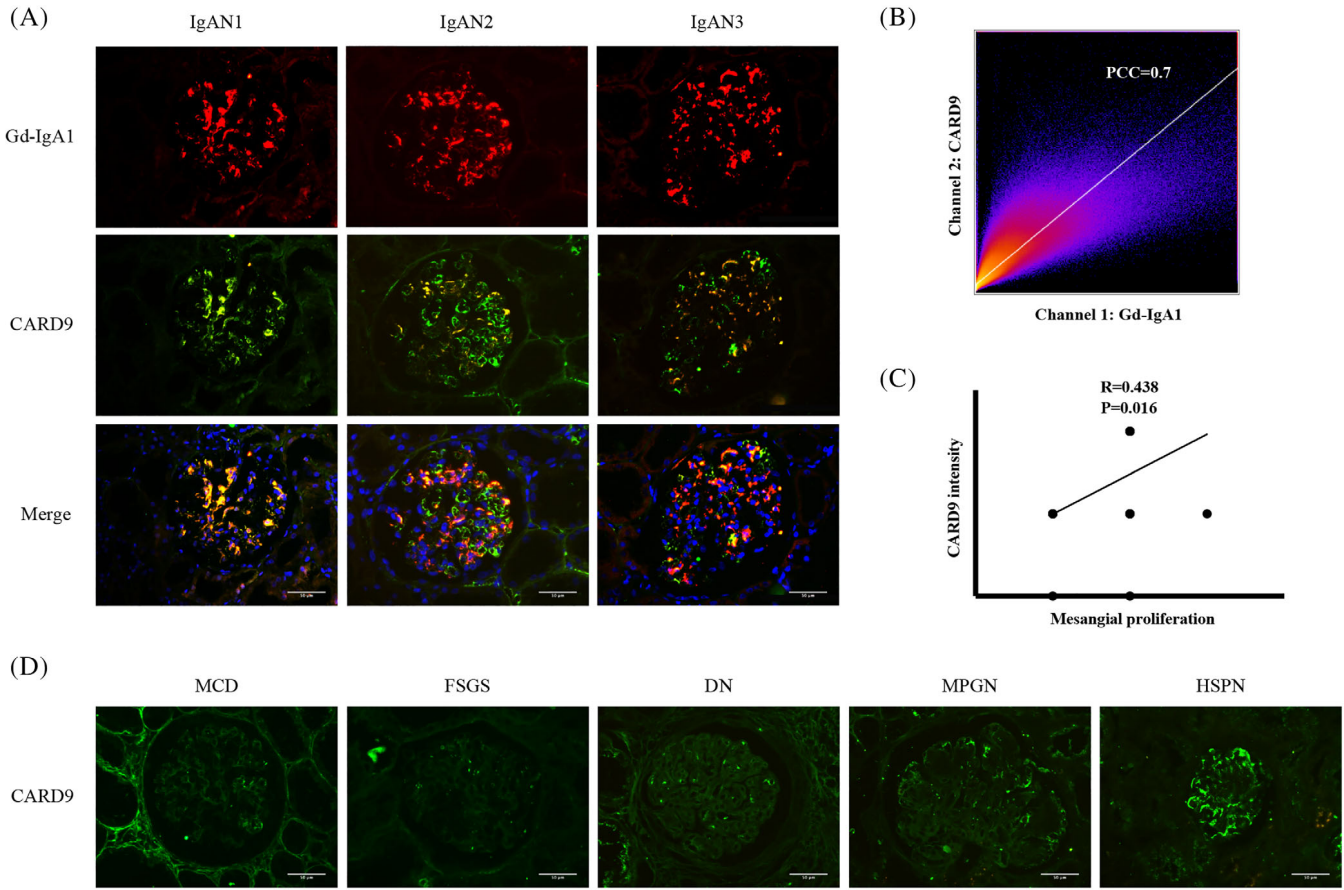
CARD9 positively correlates with IgAN mesangial proliferation. (A) Double immunofluorescence of Gd‐IgA1 and CARD9 in IgAN patients. (B) Colocalization analysis of Gd‐IgA1 and CARD9 in IgAN patients (PCC = 0.7). (C) Correlation analysis between CARD9 intensity and mesangial proliferation (*R* = 0.438, *p* = 0.016). (D) Immunofluorescence of CARD9 in patients with MCD, FSGS, DN, MPGN, and HSPN. Bars = 50 μm. All images ×400.

We verified the above results using renal biopsy specimens from our hospital. Double immunofluorescence staining of Gd‐IgA1 and CARD9 in patients with IgAN showed that CARD9 was mainly expressed in the mesangium and overlapped with Gd‐IgA1. Figure 5A shows three IgAN patients that represent the degree of overlap between CARD9 and Gd‐IgA1 as high, moderate, and low. The average Pearson's correlation coefficient (PCC) of the colocalization analysis of Gd‐IgA1 and CARD9 was 0.7, indicating a high degree of colocalization (Figure 5B). Table 2 shows the relationship between CARD9 intensity and mesangial proliferation intensity (one, mild; two, moderate; and three, severe) in 30 patients with IgAN. A significant positive correlation was observed between CARD9 intensity and mesangial proliferation, with a correlation coefficient of 0.438 (*p* < 0.05) (Figure 5C). In addition, CARD9 staining in renal biopsies with minimal change disease (MCD), focal segmental glomerulosclerosis (FSGS), diabetic nephropathy (DN), and membranoproliferative glomerulonephritis (MPGN) was negative while that of Henoch–Schonlein purpura nephritis (HSPN) was positive (Figure 5D).

**TABLE 2:** CARD9 intensity of 30 IgAN patients with different degrees of mesangial proliferation

CARD9 intensity	Mesangial proliferation		
	Mild	Moderate	Severe
0	1^a^	1	0
1+	7	7	1
2+	0	13	0

^a^ The number of patients.

### 3.5 TP regulated HMC autophagy and proliferation by CARD9

**FIGURE 6:**
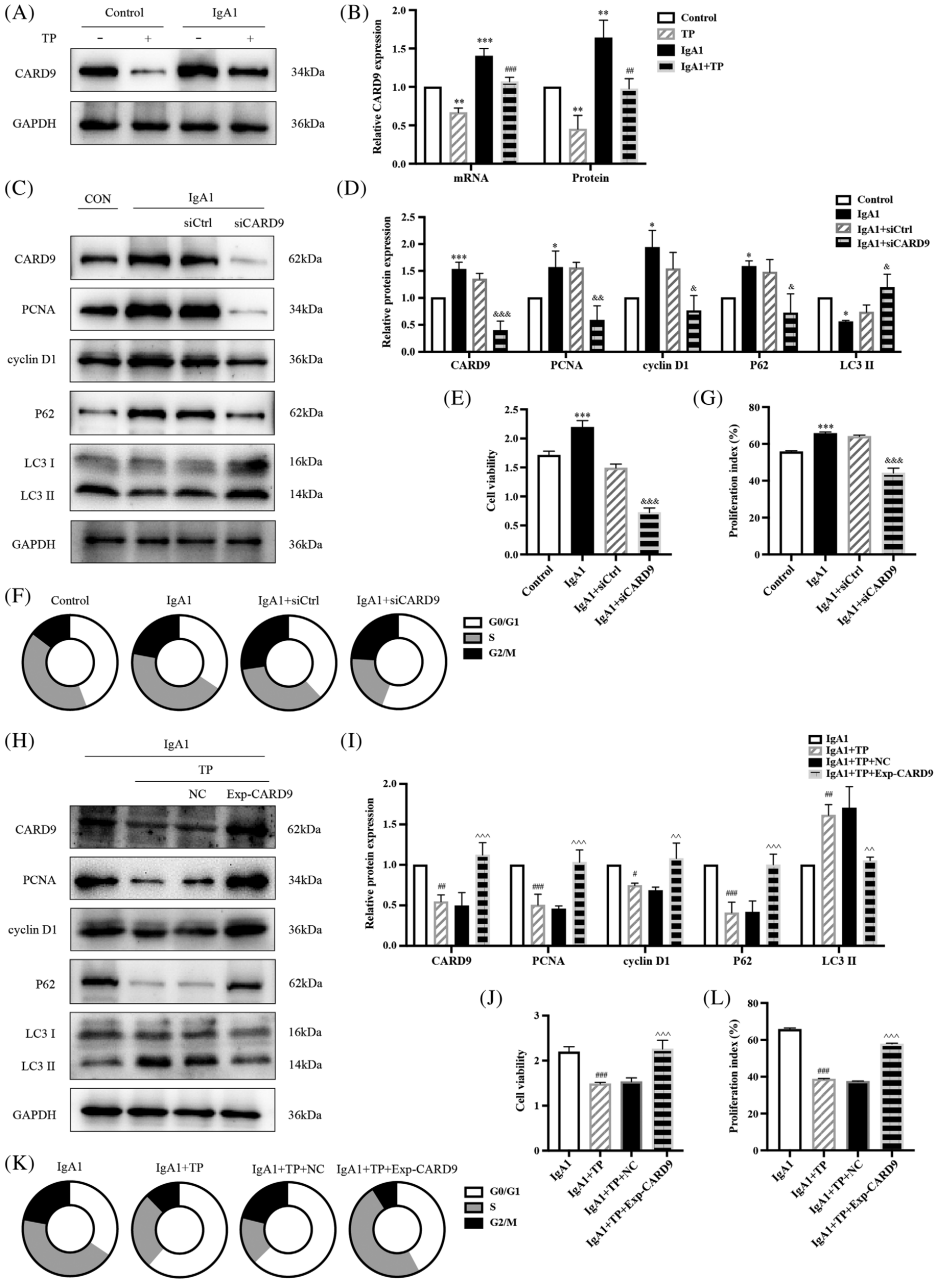
TP regulates HMC autophagy and proliferation by CARD9. Representative western blots of CARD9 (A) and quantitative analysis of CARD9 mRNA and protein levels (B). Representative western blots (C) and quantitative analysis (D) of PCNA, cyclin D1, P62, and LC3 II in CARD9‐knockdown HMCs. (E) Cell viability was assessed by CCK8 assay. (F,G) Flow cytometry analysis of cell cycle. The proliferation index is calculated as the sum of the percentages of cells in S phase and G2/M phase. Representative western blots (H) and quantitative analysis (I) of PCNA, cyclin D1, P62, and LC3 II in CARD9‐overexpression HMCs. (J) Cell viability was assessed by CCK8 assay. (K,L) Flow cytometry analysis of cell cycle. All data presented as mean ± SD, *n* = 3. **p < 0.01, **p < 0.01, ***p < 0.001 vs. Control; ^#^ *p* < 0.05, ^##^ *p* < 0.01, ^###^ *p* < 0.001 vs. IgA1; ^&^ *p* < 0.05, ^&&^ *p* < 0.01, ^&&&^ *p* < 0.001 vs. IgA1 + siCtrl; ^^^ *p* < 0.05, ^^^^ *p* < 0.01, ^^^^^ *p* < 0.001 vs. IgA1 + TP + NC.

Consistent with the sequencing results (Figure S2), the CARD9 mRNA and protein levels increased in IgA1‐induced HMCs. TP reduced the expression of CARD9 (Figure 6A,B). IF showed that the high expression of CARD9 in the glomeruli of IgAN mice was reduced by TP treatment (Figure S3). To explore the role of CARD9 in autophagy and proliferation, we constructed CARD9 siRNA and overexpression plasmids. CARD9 knockdown downregulated PCNA, cyclin D1, and P62 and upregulated LC3 II in IgA1‐induced HMCs (Figure 6C,D). The CCK‐8 assay indicated that siCARD9 inhibited the viability of IgA1‐induced HMCs (Figure 6E). Cell cycle analysis by flow cytometry showed that IgA1 increased the percentages of cells in the S and G2/M phases, IgA1 + siCARD9 arrested cells in the G0/G1 phase (Figure 6F), and CARD9 knockdown reduced the proliferation index of IgA1‐induced HMCs (Figure 6G).

In addition, we transfected a CARD9 overexpression plasmid into IgA1‐induced HMCs. The CCK‐8 assay showed that CARD9 overexpression promoted cell viability (Figure S4A). However, compared to IgA1 + NC, significant differences were not observed in the protein levels of proliferation and autophagy indicators (Figure S4B,C). Notably, CARD9 blocked the antiproliferative effect of TP on IgA1‐induced HMCs. CARD9 overexpression upregulated PCNA, cyclin D1, and P62, and downregulated LC3 II in IgA1 + TP HMCs (Figure 6H‐I). The CCK‐8 assay also showed that CARD9 restored the viability of IgA1 + TP HMCs (Figure 6J). A cell cycle analysis by flow cytometry showed that IgA1 + TP arrested cells at the G0/G1 phase, IgA1 + TP + Exp‐CARD9 increased the percentages of cells in the S and G2/M phases (Figure 6K), and CARD9 overexpression upregulated the proliferation index of HMCs with TP (Figure 6L). All results indicated that CARD9 upregulation inhibited autophagy and promoted the proliferation of HMCs; thus, TP suppressed HMC proliferation by inhibiting CARD9.

### 3.6 TP regulated HMC autophagy and proliferation via the CARD9/p38 MAPK pathway

**FIGURE 7:**
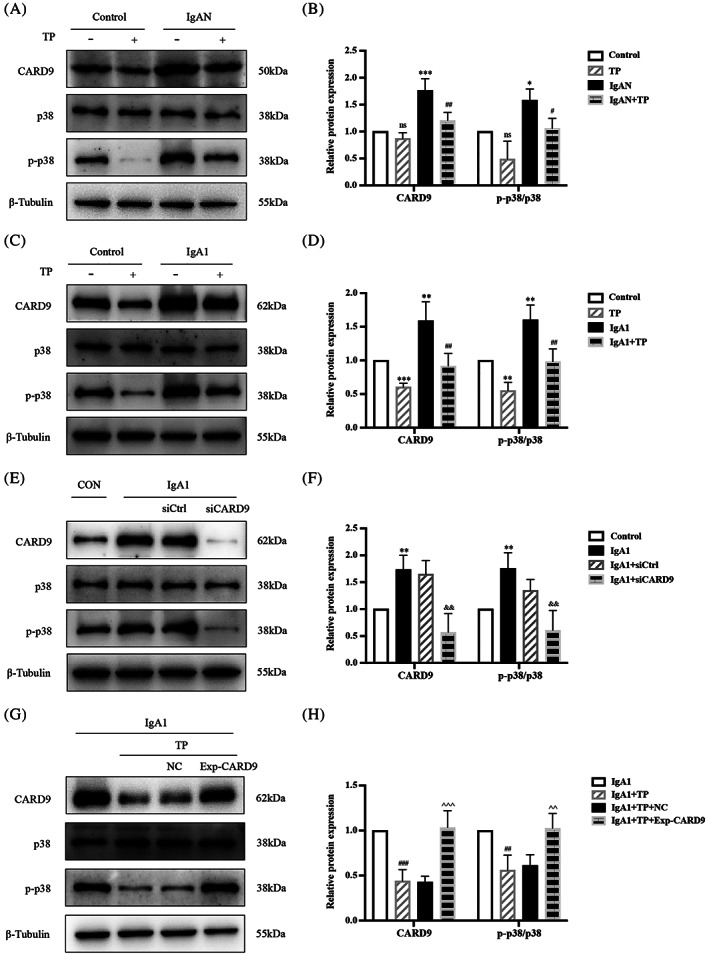
TP regulates HMC autophagy and proliferation via the CARD9/p38 MAPK pathway. Representative western blots of CARD9, p‐p38 MAPK, and p38 MAPK in (A) mice, (C) HMCs, (E) CARD9‐knockdown HMCs, and (G) CARD9‐overexpression HMCs. (B, D, F, H) Quantitative analysis of CARD9 and p‐p38 MAPK/p38 MAPK. All data presented as mean ± SD, *n* = 3. ns: *p* > 0.05 vs. Control, *p < 0.05, **p < 0.01, ***p < 0.001 vs. Control; ^#^ *p* < 0.05, ^##^ *p* < 0.01, ^###^ *p* < 0.001 vs. IgAN/IgA1; ^&&^ *p* < 0.01 vs. IgA1 + siCtrl; ^^^^ *p* < 0.01, ^^^^^ *p* < 0.001 vs. IgA1 + TP + NC.

p38 MAPK is a transcription factor regulated by CARD9. Figure 7 shows that the CARD9 and p‐p38 MAPK/p38 MAPK ratios were significantly increased in IgAN mice. After TP treatment, the levels of CARD9 and p‐p38 MAPK/p38 MAPK were reduced (Figure 7A,B). Similar results were observed in IgA1‐induced HMCs, which indicated that TP regulated autophagy and proliferation of HMCs via the p38 MAPK pathway (Figure 7C‐D). To confirm that TP regulated p38 MAPK via CARD9, we determined the p‐p38 MAPK/p38 MAPK ratios after knockdown or overexpression of CARD9. CARD9 knockdown downregulated p‐p38 MAPK/p38 MAPK in IgA1‐induced HMCs (Figure 7E,F). Meanwhile, CARD9 overexpression upregulated p‐p38 MAPK/p38 MAPK in IgA1 + TP HMCs (Figure 7G,H). These results indicated that TP promoted autophagy via CARD9/p38 MAPK to inhibit HMC proliferation.

## 4 DISCUSSION

In this study, we obtained the following results: (1) Mesangial cell proliferation was increased and autophagy was suppressed in IgAN mice and IgA1‐induced HMCs; (2) TP treatment applied to IgAN mice inhibited proliferation and promoted autophagy in mesangial cells and was safe and effective; (3) TP inhibited IgA1‐induced HMC proliferation by promoting autophagy; (4) high expression of CARD9 in IgAN was positively correlated with the severity of HMC proliferation, with CARD9 knockdown promoting autophagy and inhibiting HMC proliferation; and (5) TP promoted autophagy to inhibit HMC proliferation by downregulating the CARD9/p38 MAPK pathway in IgAN.

Tripterygium preparations are widely used for the treatment of chronic kidney disease, rheumatic and autoimmune disease, and tumors because of their immunosuppressive and anti‐inflammatory medicinal value.^17^, ^18^, ^19^ Since 1977, clinical practice and experimental research has demonstrated that tripterygium preparations are markedly effective for glomerulonephritis; however, their toxicity and side effects limit applications.^20^, ^21^, ^22^, ^23^ Purified monomeric triptolide has 100–200‐times higher potency than tripterygium glycosides.^26^, ^27^ In tumor treatment, targeted delivery of TP to diseased tissues has a curative effect and reduces toxicity.^56^, ^57^, ^58^, ^59^ Guo et al. targeted tripterine to mesangial cells using albumin nanoparticles to reduce proteinuria and inflammation in a rat nephritis model^60^ and found that the accumulation of drugs in non‐target organs was very low, thereby minimizing systemic toxicity. These studies indicate a direction for the future application of TP in kidney diseases. In our study, the IgAN mice that received TP treatment did not show obvious toxicity. Therefore, studies on the protective effect of TP on local kidneys and new targets and mechanisms contribute to maximizing the advantages and minimizing the side effects of TP treatment.

This study is the first to focus on TP in HMCs for IgAN. Previous studies have reported that tripterygium glycosides reduce Gd‐IgA1 production, proteinuria, and hematuria, inhibit inflammation, and protect renal function in IgAN rats.^61^, ^62^, ^63^ The production of Gd‐IgA1 decreases in the tonsil cells of IgAN patients treated with tripterygium glycosides.^64^, ^65^ Only one study reported that TP alleviated proteinuria and glomerular inflammation by downregulating renal TLR4, NLRP3, IL‐1β, and IL‐18 in IgAN rats.^66^ TP promotes autophagy and maintains homeostasis in IgA1‐induced podocytes.^67^ In diabetic nephropathy, TP alleviates renal fibrosis and mesangial cell proliferation.^68^, ^69^, ^70^ In rats with unilateral ureteral obstruction or chronic serum sickness nephritis, TP reduces renal fibrosis^71^, ^72^; while in rats with puromycin nephropathy or Heymann nephritis, TP protects podocytes by inhibiting immune‐mediated damage.^73^, ^74^

Glomerular mesangial cell proliferation is the most important pathological feature in the early stages of IgAN; however, its underlying mechanisms have not yet been clarified. Our previous studies suggested that autophagy is involved in HMC proliferation.^11^, ^12^ Autophagy is suppressed in IgAN rats and IgA1‐induced HMCs, and autophagy inhibitors, such as rapamycin and dihydroartemisinin, reduce HMC proliferation by inhibiting the mTOR/S6K1 pathway. However, to date, research on autophagy in IgAN has been very superficial and only focused on detecting the levels of P62 and LC3B proteins. In this study, the mRFP‐GFP‐LC3 plasmid was first used to detect the autophagic flux rate of IgAN according to the gold standards for autophagy.^75^ Quantitatively, the autophagy flux rate of HMCs decreased from 20% to 6% by IgA1, whereas TP increased it to 54%. In addition, we used the autophagy inhibitor 3‐MA to demonstrate that TP inhibited HMC proliferation by promoting autophagy. Kidney cells depend on basal autophagy for normal functioning. Consistently, increased autophagy inhibits kidney cell proliferation in diabetic nephropathy, polycystic kidney disease, renal fibrosis, and kidney cancer.^76^, ^77^, ^78^, ^79^ TP induces autophagy to regulate cell proliferation, apoptosis, differentiation, invasion, and immune response in epidural fibrosis, glioma, leukemia, pancreatic, breast, and lung cancer.^28^, ^29^, ^30^, ^31^, ^32^, ^33^

**FIGURE 8:**
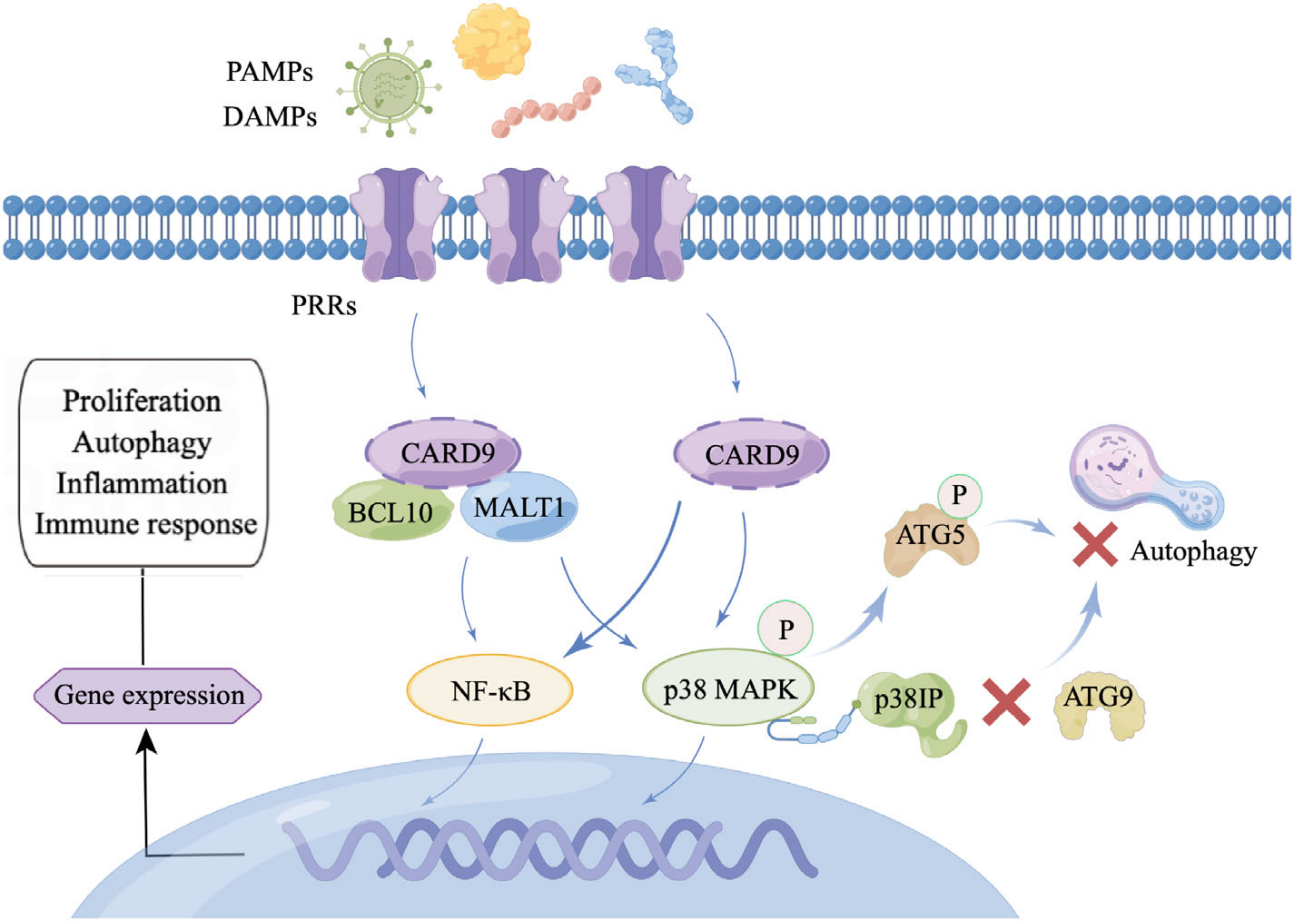
Physiological functions of CARD9 signaling. DAMPs and PAMPs sensed by PRRs lead to CARD9 activation. Next, CARD9 recruits BCL10 and MALT1 to form the CBM complex for the activation of MAPK and NF‐κB. PRRs can also directly activate CARD9. Finally, the activation of MAPK and NF‐κB contributes to gene expression related to cell proliferation, autophagy, apoptosis, differentiation, and immune response. In this interaction network, the preferential binding of p‐p38 MAPK to p38IP isolated p38IP from ATG9, thereby indirectly impairing ATG9 transport and autophagosome formation. Besides, p‐p38 MAPK‐mediated ATG5 phosphorylation inhibits autophagosome‐lysosome fusion, leading to autophagy suppression.

Our study is the first to investigate the role of CARD9 in kidney disease. CARD9 is an intracellular adaptor protein of the CARD protein family that has been identified for its selective binding to the CARD domain of B‐cell leukaemia‐lymphoma 10 (BCL10) (Figure 8). CARD9 acts as a scaffold protein. Activated CARD9 binds to BCL10 and mucosa‐associated lymphoma translocation protein 1 (MALT1) to form the CARD9/BCL10/MALT1 (CBM) complex domain, which delivers Toll‐like receptor (TLR) and C‐type lectin receptor (CLR) signals to mitogen‐activated protein kinase (MAPK) and transcription factor nuclear factor‐κB (NF‐κB).^37^, ^38^, ^39^ Therefore, CARD9 is the link between innate and adaptive immunity and has been further studied in microbial infections, especially invasive fungal infections.^80^, ^81^ CARD9 acts as a therapeutic target for cardiovascular diseases by regulating autophagy. In CARD9‐knockout mice with transverse aortic constriction, NF‐κB/p65 was inhibited to enhance autophagy and alleviate pathological cardiac remodeling.^40^ In high‐fat diet‐induced obese mice, CARD9 knockout improved obesity‐related myocardial dysfunction by reducing macrophage infiltration, inhibiting p38 MAPK phosphorylation, and enhancing autophagy.^41^ The above studies showed that inhibiting the high expression of CARD9 in the heart promotes autophagy to improve myocardial dysfunction in chronic diseases. However, CARD9 may have protective effects in acute injury models. In myocardial ischemia/reperfusion injury, CARD9 protects the myocardium by interacting with the autophagy negative regulator Rubicon to restore autophagic flux.^82^ The discrepancy between the results might be related to differences in the disease environments.

mTOR and class III PI3K complexes act in the early stages of autophagy, but little is known about their regulation in later stages. Henson et al. found that p38 MAPK inhibited autophagy independently of mTOR.^83^ The preferential binding of p‐p38 MAPK instead of autophagy protein 9 (ATG9) to p38 interacting protein (p38IP) regulates the transport and function of ATG9. Active p‐p38 isolated p38IP from ATG9, thereby indirectly impairing ATG9 transport and autophagosome formation. Blocking p38 MAPK to enhance the interaction between p38IP and ATG9 independently induces autophagy. Keil et al. also suggested that p38‐mediated autophagy protein 5 (ATG5) phosphorylation inhibits upstream of autophagosome‐lysosome fusion, thus leading to autophagy suppression.^84^ The ATG5 binding system is also a classic autophagy mechanism independent of ATG9. Consistent with our study, inhibition of the p38 MAPK pathway promotes autophagy in a variety of diseases, including Parkinson's disease, intestinal fibrosis, dry eye, macular degeneration, and gastrointestinal tumors.^43^, ^44^, ^45^, ^46^, ^47^ Our previous studies have implicated mTOR‐related autophagy in the pathogenesis of IgAN.^11^, ^12^ In this study, we demonstrated that the p38 MAPK pathway, which is regulated by CARD9, is at least partially involved in the autophagy of IgA1‐induced HMCs (Figure 8).

In conclusion, this study investigated the therapeutic effects of TP on IgAN and the important role of CARD9 in mesangial cell autophagy and proliferation. TP downregulated CARD9 to suppress p38 MAPK phosphorylation, thereby inhibiting HMC proliferation by promoting autophagy. Moreover, the findings provide new theoretical bases for mesangial cell proliferation and suggests new ideas for future targeted therapy of IgAN.

## AUTHOR CONTRIBUTIONS

The results presented in this article have not been published previously in whole or part. Conceived and designed the experiments: Lu Zhao, Hong Liu, Chengyuan Tang. Performed the experiments: Lu Zhao, Zhixin Lan, Lili Wan. Analyzed the data: Liang Peng, Xia Tan, Di Liu, Guochun Chen. Wrote the paper: Lu Zhao.

## CONFLICT OF INTEREST

The authors declare no conflict of interest.

## Supporting information


**FIGURE S1** The optimal concentration of TP in HMCs. (A) Cell viability was assessed by CCK8 assay. All data presented as mean ± SD, *n* = 3. *p < 0.05, **p < 0.01 vs. Control. (B) Representative western blot of PCNA, cyclin D1, and P62 in different concentrations of TP‐treated HMCs.
**FIGURE S2**. CARD9 is the differential gene in the dataset GSE116626 (11 control and 37 IgAN samples). (A) Heat map of the top 30 differential genes. (B) The level of CARD9 mRNA in IgAN patients compared to control (*p* < 0.05, log2 [FC] = 0.7).
**FIGURE S3**. Immunofluorescence of CARD9 in glomeruli of mice. Bars = 50 μm. All images ×400.
**FIGURE S4**. CARD9 overexpression in IgA1‐induced HMCs. (A) Cell viability was assessed by CCK8 assay. Representative western blots (B) and quantitative analysis (C) of PCNA, cyclin D1, P62, and LC3 II in CARD9‐overexpression HMCs. All data presented as mean ± SD, *n* = 3. *p < 0.05, **p < 0.01 vs. Control, ^###^ *p* < 0.001 vs. IgA1 + NC, ns: *p* > 0.05 vs. IgA1 + NC.

## Data Availability

I confirm that I have included a citation for available data in my references section. The data that support the findings of this study are available from the corresponding author upon reasonable request.
