# Supplementary figures and images for: Triptolide promotes autophagy to inhibit mesangial cell proliferation in IgA nephropathy via the CARD9/p38 MAPK pathway
Source: Cell Prolif. 2022 Jun 22;55(9):e13278. doi: 10.1111/cpr.13278 (PMC9436901; doi:10.1111/cpr.13278)

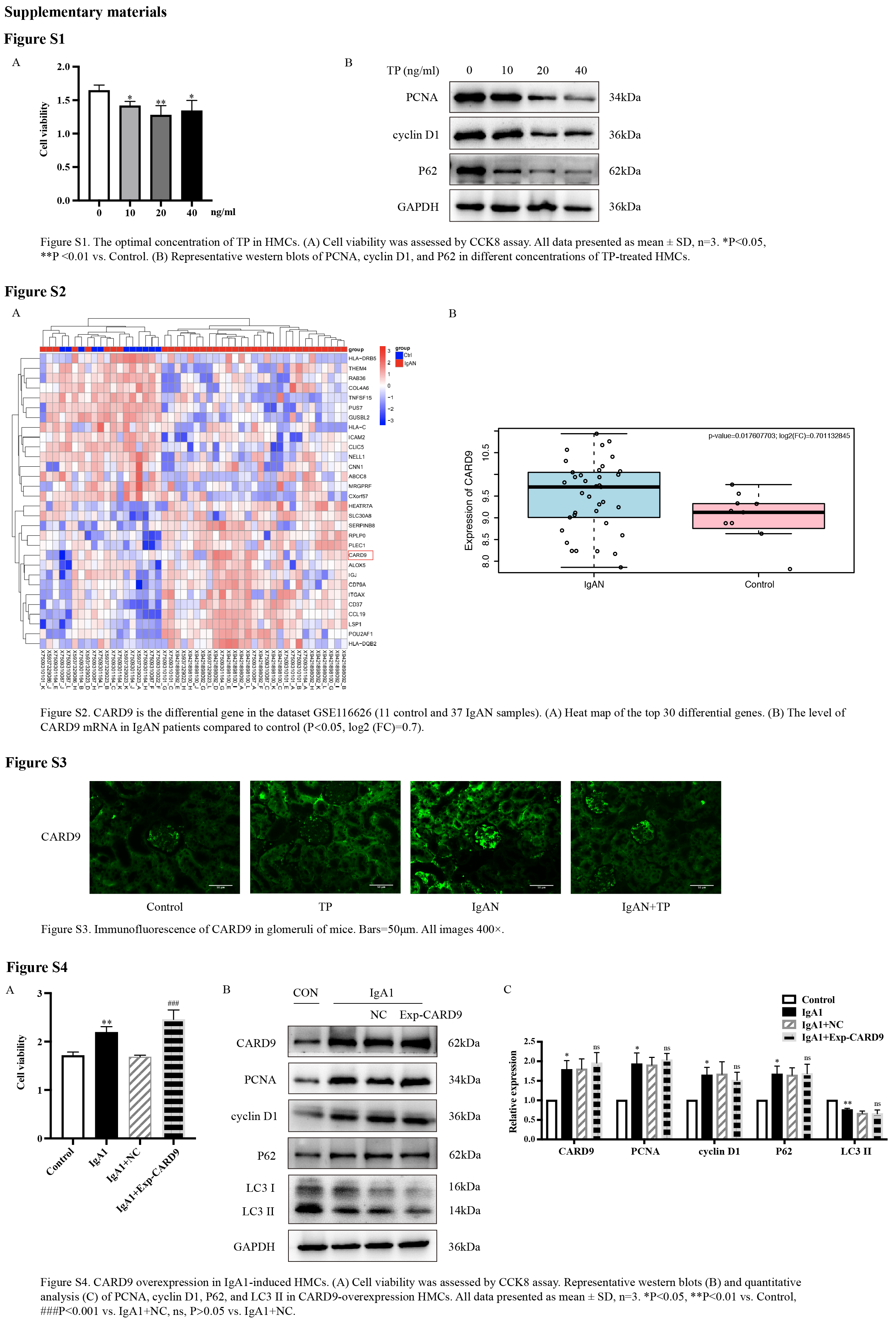

Supplement: Supplementary file 1 — FIGURE S1 The optimal concentration of TP in HMCs. (A) Cell viability was assessed by CCK8 assay. All data presented as mean ± SD, n = 3. *p < 0.05, **p < 0.01 vs. Control. (B) Representative western blot of PCNA, cyclin D1, and P62 in different concentrations of TP‐treated HMCs. FIGURE S2. CARD9 is the differential gene in the dataset GSE116626 (11 control and 37 IgAN samples). (A) Heat map of the top 30 differential genes. (B) The level of CARD9 mRNA in IgAN patients compared to control (p < 0.05, log2 [FC] = 0.7). FIGURE S3. Immunofluorescence of CARD9 in glomeruli of mice. Bars = 50 μm. All images ×400. FIGURE S4. CARD9 overexpression in IgA1‐induced HMCs. (A) Cell viability was assessed by CCK8 assay. Representative western blots (B) and quantitative analysis (C) of PCNA, cyclin D1, P62, and LC3 II in CARD9‐overexpression HMCs. All data presented as mean ± SD, n = 3. *p < 0.05, **p < 0.01 vs. Control, ### p < 0.001 vs. IgA1 + NC, ns: p > 0.05 vs. IgA1 + NC. [file CPR-55-e13278-s001.png]
